# Compression^2^: compressed sensing with compressed coil arrays

**DOI:** 10.1186/1532-429X-14-S1-P242

**Published:** 2012-02-01

**Authors:** Ganesh Adluru, Edward DiBella

**Affiliations:** 1 Radiology, University of Utah, Salt Lake City, UT, USA

## Summary

Compressed sensing with PCA based coil compression is a promising way to obtain high quality radial perfusion images without incurring the high computation cost and memory requirements associated with large multi-coil arrays

## Background

Imaging with large coil arrays is desirable for rapid imaging and high signal to noise ratio. Compressed sensing (CS) is a promising way to accelerate myocardial perfusion imaging [1]. However with increasing number of coils CS is costly in terms of memory and computation time. Coil compression methods for reconstructing cardiac cine data with parallel imaging have been proposed [2,3]. Unlike previous methods, here we employ a coil compression method with a CS reconstruction combined with a coil-based streak suppression method. The approach is tested on undersampled radial myocardial perfusion data.

## Methods

Dynamic cardiac perfusion data at rest and stress were acquired using a 32-channel cardiac coil on a Siemens 3T scanner using a 2D radial turboFLASH sequence [1]. The acquisition parameters were TR=2.4 ms, TE=1.26 ms, flip angle=12°, acquisition matrix=144x24, 0.075 mmol/kg. An ungated acquisition [4] was used, with four slices continuously acquired after each saturation pulse. 250 time frames in ~1 min. with golden ratio based angle spacing [5] between rays were acquired. The k-space data from each of the 32 coils, including all of the time frames and all of the slices, was vectorized into one column per coil and PCA was performed on the 32-column matrix. Five principal components capturing most of the variance in the data were input to the CS reconstructions.

CS reconstruction using temporal and spatial total variation constraints (STCR) [1] was performed with two GPUs using jacket [6] and parallel computing toolbox in Matlab on the five channel data as well as the 32 channel data for comparison. In order to further suppress signal from mostly streaky coils the reconstructed individual coil images were weighted by their coil sensitivities as described in [7].

## Results

Figure 1 shows 32-channel images and the five compressed coil images. Five compressed coil images are sufficient for accurate reconstruction, as seen in Figure 2 which compares CS reconstructions on the original and compressed channel data. With coil sensitivity weighting, streaks (arrows in Fig. 2) are reduced for original and compressed channel images. Images and time curves from compressed coils match well with those from all 32 coils. Reconstruction time for four slices and 250 time frames using compressed channels took ~26 min and was ~6 times longer for the 32-channel data.

## Conclusions

Compressed sensing with coil compression is a promising and efficient way to obtain high quality cardiac perfusion images using large coil arrays.

## Funding

Ben B. and Iris M. Margolis Foundation.
